# Microglial repopulation restricts ocular inflammation and choroidal neovascularization in mice

**DOI:** 10.3389/fimmu.2024.1366841

**Published:** 2024-04-22

**Authors:** Yinting Song, Yuefeng Liao, Tong Liu, Yanxian Chen, Fei Wang, Zixia Zhou, Weili Zhang, Jinying Li

**Affiliations:** ^1^ Department of Ophthalmology, Peking University Shenzhen Hospital, Shenzhen Peking University-The Hong Kong University of Science and Technology Medical Center, Shenzhen, Guangdong, China; ^2^ Experimental Ophthalmology, School of Optometry, The Hong Kong Polytechnic University, HongKong, Hong Kong SAR, China

**Keywords:** age-related macular degeneration (AMD), microglial repopulation, PLX3397, choroidal neovascularization, inflammation

## Abstract

**Introduction:**

Age-related macular degeneration (AMD) is a prevalent, chronic and progressive retinal degenerative disease characterized by an inflammatory response mediated by activated microglia accumulating in the retina. In this study, we demonstrate the therapeutically effects and the underlying mechanisms of microglial repopulation in the laser-induced choroidal neovascularization (CNV) model of exudative AMD.

**Methods:**

The CSF1R inhibitor PLX3397 was used to establish a treatment paradigm for microglial repopulation in the retina. Neovascular leakage and neovascular area were examined by fundus fluorescein angiography (FFA) and immunostaining of whole-mount RPE-choroid-sclera complexes in CNV mice receiving PLX3397. Altered cellular senescence was measured by beta-galactosidase (SA-β-gal) activity and p16INK4a expression. The effect and mechanisms of repopulated microglia on leukocyte infiltration and the inflammatory response in CNV lesions were analyzed.

**Results:**

We showed that ten days of the CSF1R inhibitor PLX3397 treatment followed by 11 days of drug withdrawal was sufficient to stimulate rapid repopulation of the retina with new microglia. Microglial repopulation attenuated pathological choroid neovascularization and dampened cellular senescence in CNV lesions. Repopulating microglia exhibited lower levels of activation markers, enhanced phagocytic function and produced fewer cytokines involved in the immune response, thereby ameliorating leukocyte infiltration and attenuating the inflammatory response in CNV lesions.

**Discussion:**

The microglial repopulation described herein are therefore a promising strategy for restricting inflammation and choroidal neovascularization, which are important players in the pathophysiology of AMD.

## Introduction

Age-related macular degeneration (AMD) is the principal cause of irreversible blindness in older individuals, worldwide (1). Currently, AMD is a devastating disease without highly effective pharmacological treatment, thus, novel therapeutic strategies are urgently needed (2, 3). Choroidal neovascularization (CNV) is a characteristic of neovascular AMD and accounts for most cases of AMD-related severe vision loss (1). The pathogenesis of neovascular AMD is multifactorial, with a combination of age, systemic health, genetic abnormalities and environmental insults involved (4). Recent information suggests that immune cells are pivotal players steering the progression of AMD (4, 5).

Microglia are the primary resident immune cells of the retina, where they act as responders to inflammation, infection, and injury (6). Microglia infiltrate the retina during embryonic development and participate in the clearance of redundant developmental neuronal cells (6). In adult eyes, microglia are mainly distributed in the ganglion cell layer, the inner plexiform layer, and the outer plexiform layer (7). Resident microglia have a highly ramified morphology allowing them to perform elaborate surveillance of their surrounding neural tissue (8). Any perturbations in the retinal microenvironment trigger microglia to lose their homeostatic signature, become activated, and subsequently migrate to the injured site (6, 8). Microglia then secrete a series of inflammatory cytokines, scavenge pathogens and phagocytose cellular debris, ultimately maintaining tissue homeostasis. Despite the protective roles that microglia play in the retina, they are negatively implicated in AMD. Studies have demonstrated that microglial accumulation in retinal degeneration sites hastens drusen formation and photoreceptor degeneration (9, 10). In addition, activated microglia can induce the release of proangiogenic factors that provide a favorable environment for neovascularization (11). Furthermore, uncontrolled and overactivated microglia can provoke the amplification of inflammation and the long-lasting destruction of retinal function (6). Consequently, finding approaches to manipulate and resolve microglial responses is an urgent matter.

Microglia are heavily reliant on signaling through colony stimulating factor 1 receptor (CSF1R) for their survival, maintenance and proliferation (12). Recently, it has been indicated that administration of small molecule CSF1R inhibitors leads to rapid and extensive elimination of microglia for the duration of treatment in the brain (12, 13). This phenomenon was also found in the retina, where microglial numbers steeply declined 24 h after CSF1R inhibition and were completely ablated after CSF1R inhibition administration for 7 days and thereafter (14). Moreover, microglia repopulated the whole retina within a relatively short period after removal of CSF1R inhibition. Repopulation is derived from both residual microglia in the optic nerve and macrophages in the ciliary body (14). PLX3397 is a novel, orally bioavailable, small molecule CSF1R inhibitor and is capable of crossing the blood retinal barrier (BRB) rapidly (15). PLX3397 has shown high efficacy in microglial depletion, providing a clinically feasible approach to achieve microglial repopulation. Recent studies have indicated that CSF1R inhibitor-dependent microglial repopulation restores classically primed microglia to homeostatic microglia (16, 17). Therefore, withdrawal of CSF1R inhibitors in the injury site resets reactive microglia and offers a possibility to promote functional recovery caused by dysfunctional microglia. Regenerated microglia promote brain recovery in CNS neurodegenerative diseases, trauma, and aging (18–21). Whether and how repopulated microglia contribute to retinal repair in degenerative retinal disease such as AMD is unknown.

In this study, we analyzed the impact of microglial repopulation in age-related macular degeneration using a mouse model of laser-induced choroidal neovascularization, and we also investigated the potential mechanisms by which regenerated microglia rescue AMD-associated deficits.

## Materials and methods

### Animals

All animal experiments were performed in accordance with animal protocols approved by the Institutional Animal Care and Use Committee of Shenzhen TopBiotech Corporation. Adult male C57BL/6J mice (6-8 weeks) were purchased from Zhuhai BesTest Bio-Tech Corporation (Zhuhai, China). Mice were housed under a 12 hr reversed light/dark cycle with ad libitum access to food and water.

### Mouse laser-induced CNV model

The mouse laser-induced CNV model was generated as previously described (22). Mice were anesthetized by ketamine/xylazine intraperitoneal injection and pupils were dilated with topical 1% tropicamide (Santen, Osaka, Japan). At the time of laser exposure, the rupture of Bruch’s membrane was confirmed by bubble formation. Four laser lesions were induced in four quadrants of each eye at approximately the same distance around the optic nerve using a slit lamp delivery system and a glass coverslip as a contact lens. Laser photocoagulation shots that cause hemorrhage or fail to produce a bubble were excluded from calculation. In the animal experiments, the examiner responsible for CNV model establishment was blinded to the animal grouping.

### Compound and drug administration

PLX3397 (Selleckchem Inc, Houston, TX) was incorporated into AIN-76A standard chow (Guangzhou Biopike Co. Ltd., Guangzhou, China). Mice were treated with PLX3397 (290 mg/kg chow) for 7 days to deplete microglia (23), followed by withdrawal for 11 days, resulting in microglial repopulation. Mice in the control groups were provided ad libitum access to standard chow.

### Fundus fluorescein angiography

Fundus fluorescein angiography (FFA) in mice was performed according to a previous study (24). Mice were anesthetized by ketamine/xylazine intraperitoneal injection and pupils were dilated with topical 1% tropicamide (Santen, Osaka, Japan). Before the procedure, 100 μL of 10% fluorescein sodium (Alcon, US) was injected intraperitoneally. We then placed the mice at a 170° angle with the head closer to the operator. Fluorescent fundus images were acquired within 5 minutes after fluorescein sodium injection. The vascular leakage area of the CNV lesions was determined by measuring the hyperfluorescent area with ImageJ Software.

### Retinal/choroid flat mount staining

For retinal/choroidal flat-mount staining, eyeballs in different groups were obtained at the different time points indicated in the figure legends. Eyes were enucleated and incubated in 4% paraformaldehyde for 1 h at room temperature and washed with PBS. The retinas or RPE-choroid-sclera complexes were dissected by removing the lens, and elements of the vitreous and optic nerves, permeabilized with 1% Triton X-100/PBS overnight at 4°C, and then blocked in PBS containing 2% BSA, and 0.3% Triton X-100. Retinas or choroids were incubated with CD31 (hamster anti-mouse CD31 monoclonal antibody, 1:120, MAB1398Z, Millipore, US), Iba-1 (rabbit anti Iba1, 1:120, 019-19741, Wako, Japan), CD68 (rat anti mouse CD68 monoclonal antibody, 1:120, MCA1957, Bio-Rad, US) or MHCII (rat anti-mouse I-A/I-E, 1:120, 556999, BD Bioscience, US) antibodies overnight at 4°C. After being washed in PBS, the tissues were incubated with the corresponding secondary antibodies (1:300, Jackson ImmunoResearch, US) for 2 h at RT. Retinas or choroids were cut partially through the sclera to the optic nerve at four places to allow the tissue to be flattened with a 4-petal shape. Images were taken with a scanning laser confocal microscope (DMI6000B with TCS SP8 system; Leica, Wetzlar, Germany) at 20× magnification in the Z-plane stacks. Images were processed using LAS X Life Science Microscope Software. The volume of choroidal neovascularization was quantified using the surface rendering tool in Imaris v9.0.1 software. The microglial number and fluorescence intensity were quantified using ImageJ software.

### Immunostaining

Eyes were fixed in 4% paraformaldehyde for 1 h at RT and then dehydrated in 30% sucrose solution overnight. Afterward, dehydrated eyes were embedded in Tissue-Tek^®^ optimum cutting temperature compound (Sakura Finetek, Japan) and cross-sectioned on a cryostat vertically through the center of the cornea and optic nerve. Serial 15-μm-thick frozen eyes sections were cut using a cryostat (NX70, Thermo Fisher Scientific, US). The eye sections were permeabilized with 0.3% Triton X-100/PBS for 30 min at RT, blocked with 2% BSA, and 0.3% Triton X-100 PBS for 1 h at RT, and then incubated at 4°C overnight with anti-CDKN2A/p16INK4a (ab211542, 1:120; Abcam, UK) and anti-CD31 (MAB1398Z, 1:120, Millipore, US) antibodies. After being washed in PBS, the sections were incubated with Alexa Fluor 488 (1:300, Jackson ImmunoResearch, US) and Alexa Fluor 594 (1:300, Jackson ImmunoResearch, US) conjugated secondary antibodies for 2 hours and 4,6-diamidino-2-phenylindole (DAPI, 1:10,000; Sigma-Aldrich, US) for 5 minutes. Images were taken with a scanning laser confocal microscope (DMI6000B with TCS SP8 system; Leica, Wetzlar, Germany). The p16^INK4a^ positive area was calculated with ImageJ software (ROI tool).

### Senescence-associated β-galactosidase staining

Senescence-associated β-galactosidase staining (SA-β-gal) was performed utilizing a senescence β-galactosidase staining kit (C0602, Beyotime, Shanghai, China) following the manufacturer’s instructions. Briefly, eye sections (15 μm) were washed with PBS and then incubated overnight at 37°C in β-galactosidase staining solution. Light microscopic pictures were acquired using an inverted fluorescence microscope (BX53, OLYMPUS, Japan).

### Phagocytosis assay and flow cytometry

For phagocytosis assay, latex beads (1 μm in diameter; L4655, Sigma) with yellow-green fluorescence (emission maximum: 515 nm) were used for phagocytosis analysis. CNV mice in the microglial repopulation and control group received an intravitreal injection of 1ul latex beads (containing 4.5×103 beads) at 13d. After 24h for intravitreal injection, the retina and RPE-choroid-sclera complex were collected and evaluated by flow cytometry.

For flow cytometry analysis of eyes, the retina and RPE-choroid-sclera complexes were collected and dissected in digest buffer containing 0.25 mg/mL DNase I (4716728001, Roche, US) and 3 mg/mL collagenase/dispase (11097113001, Roche, US). Tissue suspensions were then mechanically homogenized through a 70-μm cell strainer, washed with PBS and centrifuged at 4,500 rpm for 10 min at 4°C. Cell pellets were harvested on the bottom of the tube and suspended in ice-cold PBS for staining. The following primary antibodies directly labeled with fluorescent tags were used: anti-CD45 (1:200, 103112, Biolegend, US), anti-CD11b (1:200, 101237, Biolegend, US), anti-CD45 (1:200, 103107, Biolegend, US), anti-CD11c (1:200, 117329, Biolegend, US), anti-Ly6G (1:200, 127613, Biolegend, US), anti-Ly6C (1:200, 128007, Biolegend, US), anti-CD45 (1:200, 135523, Biolegend, US) and anti-MHC II (1:200, 107613, Biolegend, US). Dead cells were analyzed by fixable viability dye eFluor 780 (Thermo Fisher Scientific, US). Cells were fixed and permeabilized for intracellular staining with CD68 (1:200, 137013, Biolegend, US). Flow cytometry was conducted on CytoFLEX LX flow cytometer and data were analyzed by Flow Jo_V10 software.

### RNA extraction and qRT−PCR

Total RNA was extracted using TRIzol reagent (Sigma-Aldrich, MO, US) according to the manufacturer’s protocol. RNA concentrations were assessed with a NanoDrop One C spectrophotometer (Thermo, CA, US). Total RNA (1000 ng) was reverse-transcribed to cDNA using TransScript One-Step gDNA Removal and cDNA Synthesis SuperMix (Transgene, Beijing, China). qRT−PCR was performed in triplicate as 10 μL reactions for each sample using PerfectStart Green qPCR SuperMix (Transgene, Beijing, China). Amplifications were conducted using a LightCycler 480 Real-Time PCR system (Roche Diagnostics, Rotkreuz, Switzerland) with the following cycle conditions: 94°C for 30 s, 40 cycles of 94°C for 5 s, and 60°C for 30 s. The expression levels of β-actin were used as an internal control. The sequences for the real-time quantitative PCR primers are listed in **Table 1**.

**Table 1:** Primer sequences used for real-time RT-PCR.

Gene	Forward sequence	Reverse sequence
*Il-1β*	GTTCCCATTAGACAACTGCACT	CCGACAGCACGAGGCTTTT
*Il-6*	CAACCACGGCCTTCCCTACT	TTGGGAGTGGTATCCTCTGTGA
*Mcp-1*	GTCTGTGCTGACCCCAAGAAG	TGGTTCCGATCCAGGTTTTTA
*Tnfα*	ACCGTCAGCCGATTTGCTAT	TTGACGGCAGAGAGGAGGTT
*Cxcl10*	TGGCTGTCCTAGCTCTGTACTGT	GAGGACAAGGAGGGTGTGG
*Cxcl2*	CCCTTGGACATTTTATGTCTTCC	GACACGAAAAGGCATGACAA
*β-actin*	GGCTGTATTCCCCTCCATCG	CCAGTTGGTAACAATGCCATGT

### Elisa

The retina-choroid complexes were collected and dissected in iced PBS containing 1% protease inhibitor. The processed homogenate was subject to ultrasonic disruption for 30s, freeze-thawed in liquid nitrogen for twice and centrifuged at 12,000 rpm for 10min. The supernatant was collected and protein levels of inflammatory cytokines IL-1β, IL-6, MCP-1, TNFα, CXCL10 and CXCL2 were measured by ELISA. In brief, ELISA kits were allowed to equilibrate at room temperature for 30 min prior to cytokines quantification according to manufacturers’ instruction. ELISA kit (Sangon Biotech, Inc., Shanghai, China) was used to measure soluble extracellular levels of IL-1β (catalog no. D721017), IL-6 (catalog no. D721022), MCP-1 (catalog no. D721198), TNFα (catalog no. D721217), CXCL10 (catalog no. D721007) and CXCL2 (catalog no. D711244).

### RNA sequencing and analysis

RNA was extracted from microglia purified by magnetic bead cell sorting. Briefly, retina and RPE-choroid-sclera complex single-cell suspensions were prepared as described above, and CD11b enrichment was performed by magnetic bead cell sorting according to the manufacturer’s instructions (Miltenyi Biotech). Total RNA from sorted cells was extracted using TRIzol reagent following the manufacturer’s instructions. RNA samples were sent to the Beijing Genomics Institute (Shenzhen, China) for quantification, cDNA library preparation, RNA sequencing, and data analysis. Total amounts and integrity of RNA were assessed using the RNA Nano 6000 Assay Kit of the Bioanalyzer 2100 system (Agilent Technologies, CA, US). After the library was qualified, it was sequenced by an Illumina NovaSeq 6000. FeatureCounts v1.5.0-p3 was used to count the read numbers mapped to each gene. Then, the FPKM of each gene was calculated based on the length of the gene and reads count mapped to this gene, which normalizes gene expression by considering the effect of sequencing depth and gene transcript length at the same time. The gene set of interest was obtained from the Molecular Signatures Database (MSigDB) (http://www.broadinstitute.org/gsea/msigdb/) (GO:0002718, GOBP, regulation of cytokine production involved in immune response). Enrichment analysis based on Gene Ontology (GO) terms and KEGG was performed by Enrichr (https://maayanlab.cloud/Enrichr/) (25). Then, figure drawing and graphic display were performed by a website (www.bioinformatics.com.cn), an online platform for data analysis and visualization.

### Statistical analysis

Statistical analysis was performed using GraphPad Prism 8.0.1 software (GraphPad Software, San Diego, CA). All quantitative results are displayed as the mean ± SD. The statistical significance of differences between two groups was calculated with a parametric unpaired Student’s t test. The statistical significance of differences between three or more groups was calculated with one-way analysis of variance (ANOVA). A value of p< 0.05 was considered significant. *p < 0.05, **p < 0.01, ***p < 0.001. Statistical details are provided in the figure legends.

## Results

### Replacement of microglia in the retina of choroid neovascularization

**Figure 1:**
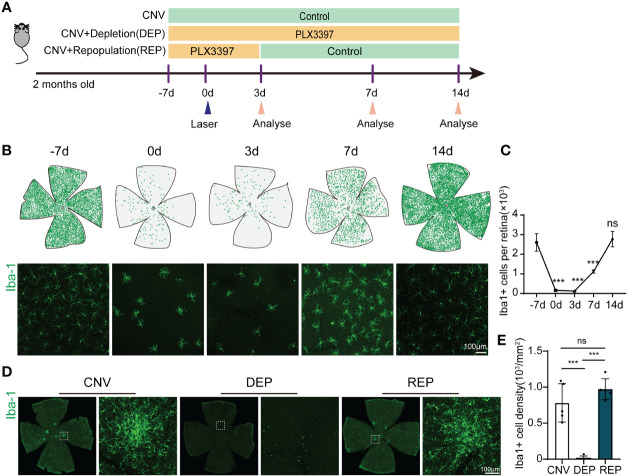
Microglial repopulation in the retina of choroid neovascularization. **(A)**. The experimental timeline illustrates drug administration and experimental design. **(B)**. Spatial distributions of retinal microglia at different time points. Each green dot represents a microglial cell. Representative confocal images of Iba-1-stained retina flat mounts at the indicated time points. **(C)**. Quantification of microglial numbers in the retinas shown in **(B)** (n=3 mice per group). **(D)**. Representative confocal images of Iba-1-stained retina flat mounts in the microglial repopulation group, microglial depletion group or control group at 14 d after laser injury. **(E)**. Quantification of microglial numbers in the retinas shown in **(C)** (n=5 mice per group). Data are presented as the mean ± SD. ns means no significance ***p < 0.001, one-way ANOVA followed by Tukey’s multiple comparisons test in **(C, E)**. Scale bars, 100 μm in **(B, D)**.

First, we tried to establish an effective treatment paradigm for microglial elimination and subsequent microglial repopulation in the retina with the CSF1R inhibitor PLX3397. We fed wild-type mice chow with PLX3397 for 10 days to eliminate microglia. Then, the mice were subjected to withdrawal of the PLX3397 formulated diet for an additional 4 days or 11 days, allowing microglia to repopulate (**Figure 1A**). We measured the number of microglia in the retinas of mice receiving PLX3397 in chow at -7 d, 0 d, 3 d, 7 d and 14 d. In accordance with prior reports (14), microglial numbers in the retina were significantly reduced after PLX3397 administration for 7 days and thereafter (-7d=2602 ± 450.6, 0d=158.3 ± 38.28, *P*<0.0001, **Figures 1B, C**). The numbers of microglia steeply increased following 4 days of drug withdrawal (-7d=2602 ± 450.6, 7d=117 ± 78.5, *P*=0.0004, **Figures 1B, C**). By eleven days of repopulation, the counts of microglia almost recovered to baseline levels (-7d=2602 ± 450.6, 14d=2773 ± 392.7, *P*=0.9324, **Figures 1B, C**). To further confirm microglial self-renewal in the CNV model, we performed laser-induced CNV after 7 days of PLX3397 treatment or normal diet treatment (intervention Day 0). In the microglia-depleted group, mice were fed PLX3397 for 21 consecutive days until sacrifice (**Figure 1A**). By 11 days of microglial repopulation, no significant differences existed in microglial number between repopulated and control CNV mice (CNV=782.4 ± 265.3, DEP=13.78 ± 30.81, REP=971.4 ± 145.6, *P*<0.0001, *P*=0.2443, *P*<0.0001, **Figures 1D, E**). Thus, ten days of PLX3397 treatment is sufficient to eliminate the overwhelming majority of microglia from the retina, while 11 days of drug withdrawal stimulates rapid repopulation of the retina with new microglia.

### Microglial repopulation attenuates pathological choroid neovascularization

**Figure 2:**
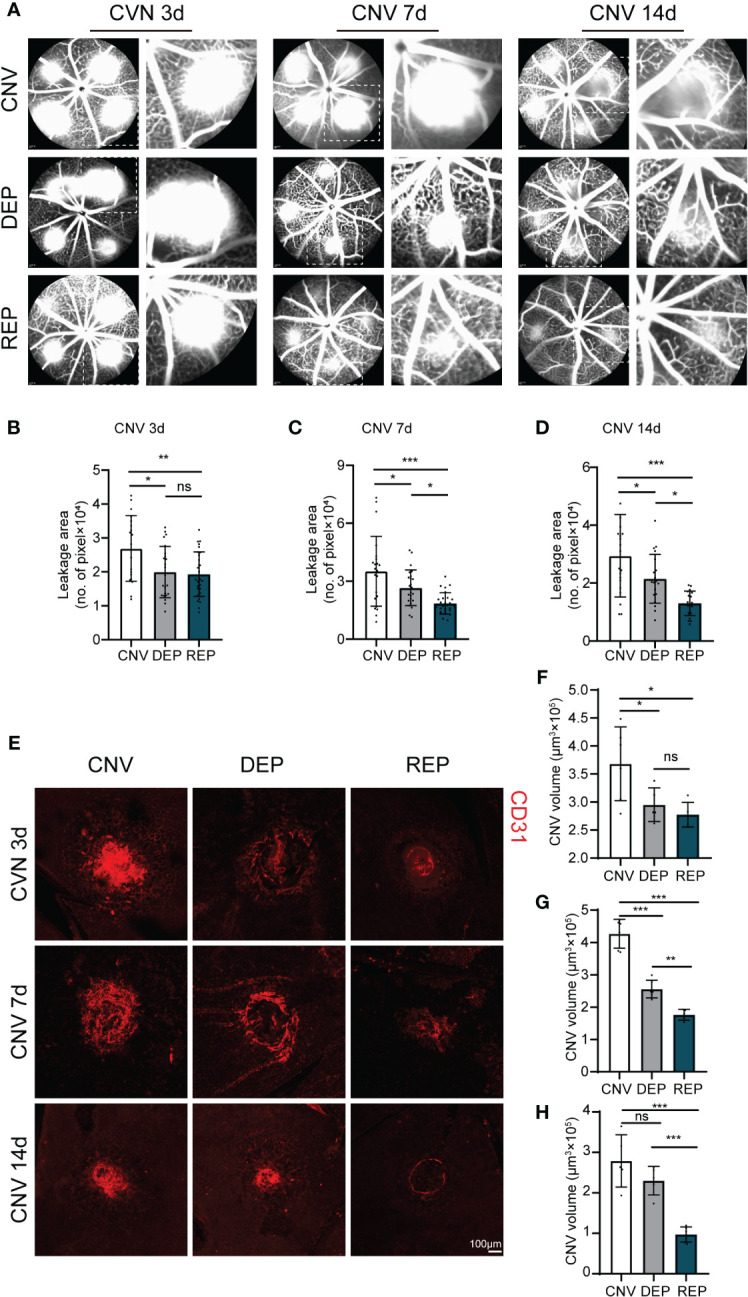
Repopulated microglia attenuate neovascular leakage and neovascular area in CNV lesions. **(A)**. Representative images of FFAs in the microglial repopulation group, microglial depletion group or control group at 3 d, 7 d or 14 d after laser injury. **(B-D)**. Quantification of the vascular leakage area shown in **(A)** (n = 19/23/29 mice per group at 3 d, n =22/24/27 mice per group at 7 d, n = 20/20/20 mice per group at 14 d). **(E)**. Representative confocal images of CD31-stained RPE-choroid-sclera flat mounts in the microglial repopulation group, microglial depletion group or control group at 3 d, 7 d or 14 d after laser injury. **(F-H)**. Quantification of CNV volume shown in **(E)** (n = 6/5/5 mice per group at 3 d, n =6/6/5 mice per group at 7 d, n = 5/5/6 mice per group at 14 d). Data are presented as the mean ± SD. ns means no significance *p < 0.05, **p < 0.01, ***p < 0.001, one-way ANOVA followed by Tukey’s multiple comparisons test in **(B-D)** and **(F-H)**. Scale bars, 100 μm in **(E)**.

To explore the potential effect of microglial repopulation on pathological choroid neovascularization, we examined neovascular leakage and neovascular area in CNV mice receiving PLX3397 in chow followed by several days of withdrawal, PLX3397 in chow consecutively, or a control diet. FFA was performed after laser injury to observe vascular leakage. Leakage area quantification showed that both microglial depletion and microglial repopulation significantly abrogated vascular leakage at 3 d, 7 d and 14 d (3d: CNV=26916 ± 9696, DEP=19967 ± 7561, REP=19316 ± 6563, *P*=0.0150, *P*=0.0044; 7d: CNV=35182 ± 18049, DEP=26606 ± 9193, REP=18513 ± 5496, *P*=0.0402, *P*<0.0001; 14d: CNV=29430 ± 14249, DEP=21518 ± 8421, REP=13017 ± 4201, *P*=0.0365, *P*<0.0001, **Figures 2A-D**). The leakage of CNV was markedly reduced in the repopulation group in comparison to the depletion group at 7 d and 14 d (*P*=0.0420, *P*=0.0227; **Figures 2A-D**). Furthermore, we determined neovascular formation by CD31 staining of whole-mount RPE-choroid-sclera complexes. Similarly, a decrease in neovascular volume was observed in the microglial depletion and microglial repopulation groups in comparison to the control group at 3 d, 7 d and 14 d (3d: CNV=368265 ± 65880, DEP=295059 ± 30028, REP=277397 ± 21931, *P*=0.0499, *P*=0.0154; 7d: CNV=426830 ± 44471, DEP=255833 ± 27474, REP=176145 ± 16776, *P*<0.0001, *P*<0.0001; 14d: CNV=279047 ± 64525, DEP=230191 ± 35222, REP=97193 ± 18686, *P*=0.2010, *P*<0.0001, **Figures 2E-H**). The CNV volume was much smaller in the repopulation group than in the depletion group at 7 d and 14 d (*P*=0.0032, *P*=0.0005; **Figures 2E-H**). There was no significant difference in the leakage area and volume of neovascularization between the depletion and repopulation groups at 3 d (*P*=0.9522, *P*=0.8172; **Figures 2E-H**), considering that the two groups of mice were treated the same before. These results indicate that microglial depletion has beneficial effects against CNV, which is consistent with results reported previously. Moreover, microglial repopulation has a better protective effect on CNV than depletion alone.

### Microglial repopulation alters microglia phagocytosis and dampens cellular senescence in CNV lesions

**Figure 3:**
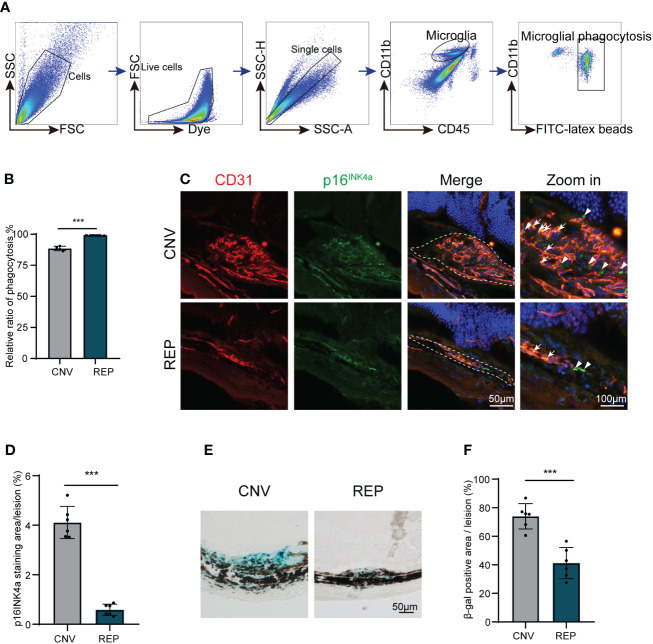
Repopulated microglia enhanced phagocytosis function and dampen cellular senescence in CNV lesions. **(A)**. Gating strategy for flow cytometry analysis of microglia phagocytic ability in the microglial repopulation or control group at 14d. **(B)**. Flow cytometry quantification of yellow-green fluorescence labeled microglial phagocytosis of latex beads (n = 4/4 from 12/12 mice per group). **(C)**. Representative images of eye sections immunostained from CNV mice in the microglial repopulation or control group at 14 d showing the p16INK4a (green)-positive area in CNV lesions. CD31 (red) was used to indicate choroidal neovascularization, and DAPI (blue) was used to stain nuclei. **(D)**. Quantification of the p16INK4a (green)-positive area in **(C)** (n=6/6 mice per group). **(E)**. Representative images of SA-β gal staining (blue) in eye cross sections from CNV mice in the microglial repopulation or control group at 14 d. **(F)**. Quantification of the SA-β gal (blue)-positive area in **(E)** (n=6/6 mice per group). Data are presented as the mean ± SD. ***p < 0.001, unpaired Student’s t test in **(B, D, F)** after testing for normality using Shapiro-Wilk test. Scale bars, 50 μm in **(C, E)**.

Phagocytosis is a major function of microglia. In response to pathological stimuli, microglia can scavenge pathogens and phagocytose cellular debris, ultimately maintaining tissue homeostasis (5). To evaluate a possible protective role of microglial repopulation, we measured phagocytic function of microglia in CNV lesions as previously reported (26, 27). Flow cytometry indicated that more latex beads were engulfed by repopulated microglia than resident microglia (CNV=88.53 ± 1.746, REP=99.25 ± 0.3109, P<0.001, **Figures 3A, B**).

Since previous studies have demonstrated that senescent cells can accelerate pathological neovascularization and that clearance of senescent cells can suppress neovascularization (28, 29). As one important microglial function is the removal and clearance of tissue debris (6), we next investigated whether cellular senescence characteristics exist in CNV lesions and whether they could be diminished by repopulated microglia. Senescence-associated beta-galactosidase (SA-β-gal) activity and p16^INK4a^ expression are important features of cellular senescence. We found that positive fluorescent signals of p16^INK4a^ (senescent cell markers) accumulated in CD31-positive areas (**Figure 3C**). Consistently, SA-β-gal-stained ocular sections revealed that SA-β-gal was abundantly present in the retina-choroid complex of CNV mice (**Figure 3E**), suggesting that senescent cells are enriched in CNV lesions. Microglial repopulation in the CNV eyes significantly reduced the SA-β-gal-positive areas (CNV=74.00 ± 8.851, REP=41.17 ± 10.91, *P*=0.0002, **Figure 3F**) and p16^INK4a^ expression (CNV=4.114 ± 0.6500, REP=0.5840 ± 0.2292, *P*<0.0001, **Figure 3D**) in CNV lesions, indicating that microglial repopulation dampens cellular senescence in CNV lesions.

These data further verify our conjecture that repopulated microglia reverse dystrophic and senescent status and show enhanced phagocytic function, thereby leading to the clearance of cellular debris and senescent cells, consequently suppress neovascularization.

### Microglial repopulation reduced CXCL2 expression and thereby suppresses leukocyte infiltration in CNV lesions

**Figure 4:**
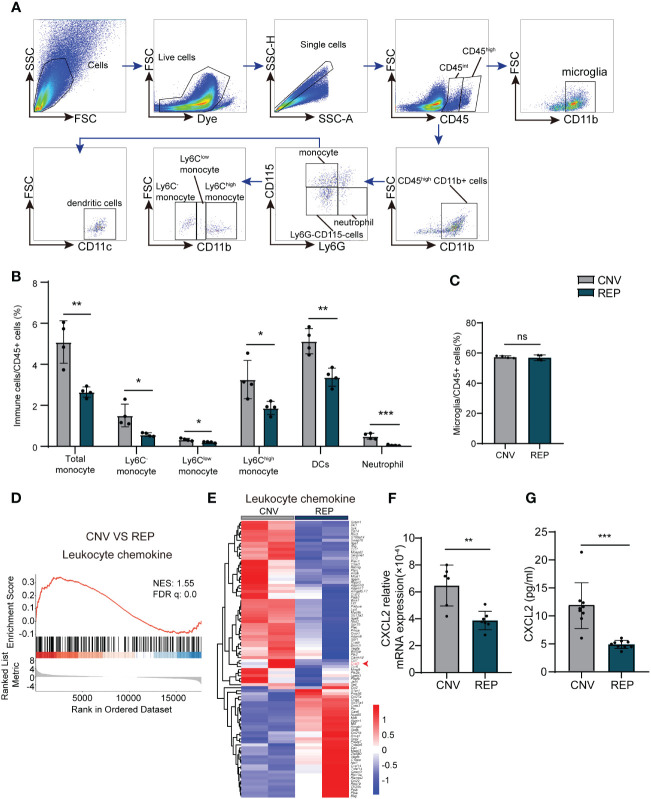
Repopulated microglia suppress the infiltration of leukocytes in CNV lesions. **(A)**. Gating strategy for flow cytometry analysis of eye-infiltrating neutrophils (CD45highCD11b+Ly6G+CD115-), total monocytes (CD45highCD11b+Ly6G-CD115+), Ly6C-monocyte (CD45highCD11b+Ly6G-CD115+Ly6C-), Ly6Chigh monocyte (CD45highCD11b+Ly6G-CD115+Ly6Chigh), Ly6Clow monocytes (CD45highCD11b+Ly6G-CD115+Ly6Clow), dendritic cells (CD45highCD11b+Ly6G-CD115-CD11c+) and microglia (CD11b+CD45int). **(B, C)**. Counts of leukocyte subsets in the retina-choroid complex from the indicated groups of CNV mice at 14 d (n=4/4 from 12/12 mice per group). **(D)**. GSEA histograms for the “Leukocyte chemokine” gene set in CNV mice with or without microglial repopulation. NES: normalized enrichment score. **(E)**. Heatmap representation of the DEGs genes associated to leukocyte chemokines in microglia in the repopulation and control groups. **(F)**. mRNA expression changes of *Cxcl2* in retinal tissue detected by RT-PCR (n = 6/7 mice per group). **(G)**. Protein expression changes of CXCL2 in retinal tissue detected by Elisa. n = 9/9 mice per group. Data are presented as the mean ± SD. ns means no significance *P < 0.5; **P < 0.1; ***P < 0.001, Mann-Whitney test in [**(B)** Ly6Clow monocyte, **(G)**] and unpaired Student’s t test in other graphs after testing for normality using Shapiro-Wilk test.

**Figure 5:**
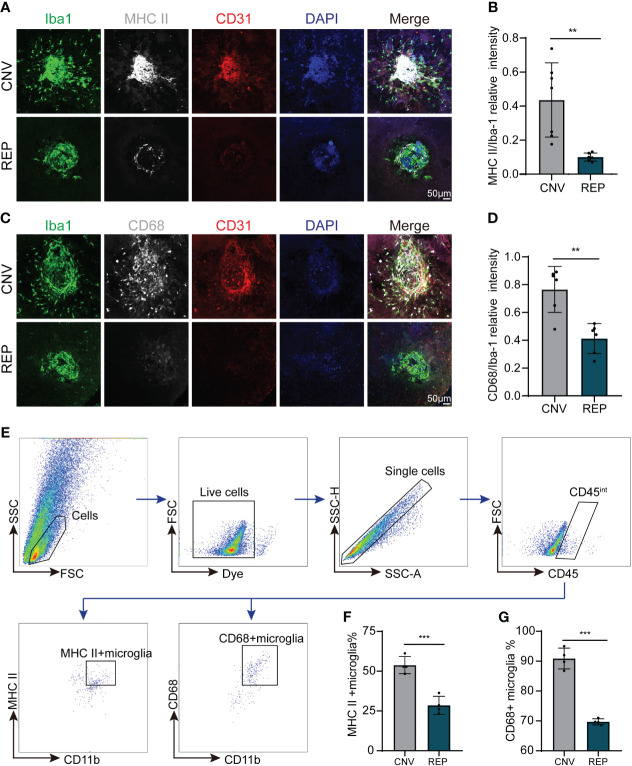
Repopulated microglia restrict the activation of microglia in CNV lesions. **(A)**. Representative confocal images of RPE-choroid-sclera flat mounts from CNV mice in the microglial repopulation or control group at 14 d showing MHC II (white) expression in Iba-1+ infiltrated microglia. CD31 (red) was used to indicate choroidal neovascularization, and DAPI (blue) was used to stain nuclei. **(B)**. Quantification of MHC II (white) expression in **(A)** (n=7/6 mice per group). **(C)**. Representative confocal images of RPE-choroid-sclera flat mounts from CNV mice in the microglial repopulation or control group at 14 d showing CD68 (white) expression in Iba-1+ infiltrated microglia. CD31 (red) was used to indicate choroidal neovascularization, and DAPI (blue) was used to stain nuclei. **(D)**. Quantification of CD68 (white) expression in **(C)** (n=6/6 mice per group). **(E)**. Gating strategy for flow cytometry analysis of microglia subpopulation in the microglial repopulation or control group at 14d. **(F, G)**. Quantification of MHC II+ microglia **(F)** (n=4/4 from 12/12 mice per group) and CD68+ microglia percentage **(G)** (n=4/4 from 12/12 mice per group) of total gated microglia. Data are presented as the mean ± SD. **p < 0.01, ***p < 0.001, unpaired Student’s t test in **(B, D)** after testing for normality using Shapiro-Wilk test.

To investigate the influence of microglial repopulation on leukocyte infiltration in the retina, we determined the counts of immune cell subsets in the retina-choroid complex of CNV mice receiving microglial repopulation by flow cytometry (**Figure 4A**). We found that neutrophils (CD45highCD11b+Ly6G+CD115-) (CNV=0.4907 ± 0.1439, REP=0.05346 ± 0.02820, *P*=0.0010, **Figure 5B**), total monocytes (CD45highCD11b+Ly6G-CD115) (CNV=5.086 ± 1.039, REP=2.653 ± 0.2545, *P*=0.0039, **Figure 5B**) and dendritic cells (CD45highCD11b+Ly6G-CD115-CD11c+) (CNV=5.124 ± 0.6172, REP=3.372 ± 0.4406, *P*=0.0036, **Figure 5B**) were markedly reduced in the lesions of CNV mice receiving microglial repopulation versus the control diet (**Figures 5A, B**). Detailed analysis revealed that PLX3397 treatment reduced the frequency of Ly6C-, Ly6Chigh and Ly6Clow monocytes (Ly6C-: CNV=1.503 ± 0.5539, REP=0.5720 ± 0.09109, *P*=0.0160; Ly6Chigh: CNV=3.255 ± 0.9349, REP=1.872 ± 0.3198, *P*=0.0312; Ly6Clow: CNV=0.3268 ± 0.06214, REP=0.2028 ± 0.02965, *P*=0.0286; **Figures 4A, B**). The flow cytometry results also revealed similar counts of microglia (CD45intCD11b+) in the retina-choroid complex between the microglial repopulation and control groups (CNV=57.38 ± 0.7714, REP=56.91 ± 1.864, *P*=0.6558; **Figure 4C**), confirming the high efficiency of repopulation. These data demonstrate that microglial repopulation suppresses the infiltration of leukocytes into CNV lesions.

To further elucidate how repopulated microglia ameliorated leukocytes infiltration in the CNV model. We have analyzed the RNA-seq data. GSEA analysis showed the significant down regulation of the leukocyte chemokine in repopulated microglia compared to resident microglia in the CNV model (**Figure 4D**). Heatmap analysis showed that the levels of transcripts involved in “leukocyte chemokine”, including C-X-C Motif Chemokine 2 (CXCL2) were decreased in the repopulated microglia of CNV mice (**Figure 4E**). CXCL2 known as pro-migratory factors can be produced by activated microglia and recruit neutrophils and other leukocytes to sites of inflammation (30). We then confirmed the RNA transcription of CXCL2 by RT-PCR (CNV=0.0006477 ± 0.0001519, REP=0.0003877 ± 0.00006879, *P*=0.0018; **Figure 4F**) and the protein expression of CXCL2 by Elisa (CNV=11.83 ± 4.094, REP=4.917 ± 0.7122, *P*<0.0001; **Figure 4G**). These results indicate that microglial repopulation reduced the expression of leukocyte-attracting chemokines CXCL2, which led to reduced leukocyte infiltration.

### Microglial repopulation restricts microglial activation in CNV lesions

As microglia have been reported to be overactivated in the process of CNV (6), we evaluated whether microglial repopulation within the eye influenced their activation status in CNV lesions by immunostaining and flow cytometry. We observed large numbers of microglia with high expression of activation markers (MHCII (H2-Aa) and CD68) recruited around the site of laser injury (**Figures 5A, C**). Notably, microglial repopulation reduced the expression of MHCII and CD68 in accumulated microglia (CNV=0.4361 ± 0.2181, REP=0.1003 ± 0.02372, *P*=0.0033, **Figure 5B**; CNV=0.7658 ± 0.1657, REP=0.4122 ± 0.1080, *P*=0.0014, **Figure 5D**). For flow cytometry, we gated microglia with CD45int and CD11b+, and then quantified the percentage of MHC II+ or CD68+ microglia to total microglia. The results showed that the percentage of MHC II+ and CD68+ microglia significantly decreased in microglial repopulation group compared to the control group (CNV=53.83 ± 5.369, REP=28.55 ± 5.653, *P*=0.0006, **Figures 5E, F**; CNV=90.90 ± 3.471, REP=69.71 ± 1.003, *P*<0.0001, **Figure 5G**). Overall, these data suggest that microglial repopulation restricts microglial activation in CNV lesions.

### Microglial repopulation reduces the production of cytokines involved in the immune response and attenuates the inflammatory response in CNV lesions

**Figure 6:**
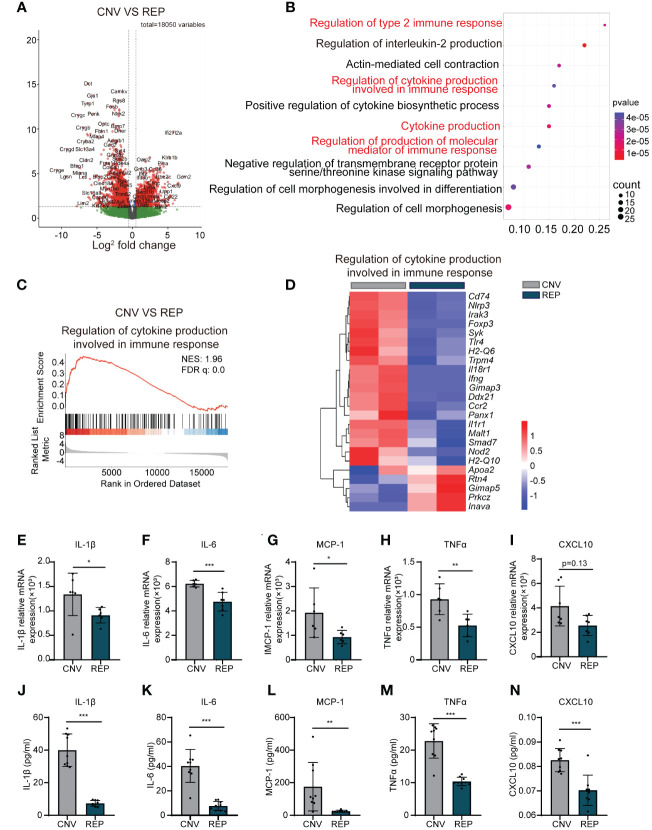
Repopulated microglia reduce the production of cytokines involved in the immune response and attenuate the inflammatory response in CNV lesions. **(A)**. A volcano plot showed the differentially expressed genes (Use log(Foldchange)>1, padj<0.05 as the difference significance criterion). **(B)**. Functional enrichment analysis using GO and KEGG for the DEGs, with the top ten pathways displayed. **(C)** GSEA histograms for the “Regulation of cytokine production involved in immune response” gene set in CNV mice with or without microglial repopulation. Enrichment plots demonstrate downregulation of cytokine production involved in the immune response in repopulating microglia compared to nonrepopulating microglia in CNV mice. NES: normalized enrichment score. **(D)** Heatmap analysis of the DEGs involved in the regulation of cytokine production involved in the immune response signaling pathway between the repopulation and control groups. **(E-I)**. mRNA expression changes of various inflammatory cytokines and chemokines, including *Il-1β* (n=6/6 mice per group), *Il-6* (n=6/7 mice per group), *Mcp-1* (n=6/6 mice per group), *Tnfα* (n=6/6 mice per group), and *Cxcl10* (n=7/7 mice per group) in retinal tissue detected by RT−PCR. **(J-N)**. Detection of cytokines including IL-1β, IL-6, MCP-1, TNF-α and CXCL10 in the retina-choroid complex tissue by ELISA (n=9/9 mice per group). Data are presented as the mean ± SD from at least 6 mice per group. *p < 0.05, **p < 0.01, ***p < 0.001, Mann-Whitney test in **(I, J)** and unpaired Student’s t test in **(E-H, K-N)** after testing for normality using Shapiro-Wilk test.

We next sought to explore the potential mechanisms through which microglial repopulation protects against CNV. We used magnetic-activated cell sorting (MACS) to isolate microglia from the retina-choroid complex of CNV mice, either repopulated or control, followed by RNA-Seq analysis of the transcriptome. The differentially expressed genes were displayed by a volcano plot (Use log(Foldchange)>1, padj<0.05 as the difference significance criterion) (**Figure 6A**). Enrichment analysis of differentially expressed genes (DEGs) using the GO and KEGG pathways highlighted a significant enrichment within the regulation of cytokine production involved in the immune response (**Figure 6B**). Gene set enrichment analysis (GSEA) further demonstrated the downregulation of the regulation of cytokine production involved in the immune response pathway in repopulating microglia compared to nonrepopulating microglia (**Figure 6C**). Heatmap analysis showed that the levels of transcripts involved in this signaling pathway were decreased in the repopulated microglia of CNV mice (**Figure 6D**). Furthermore, we assessed whether the repopulating microglia modulated the expression of cytokines and chemokines known to impact the inflammatory status of the eye. RNA was isolated and analyzed for the expression of various inflammatory mediators using specific primers (**Table 1**) by qRT−PCR. Microglial repopulation significantly reduced the expression of *Il-1β* (CNV=0.001339 ± 0.0004341, REP=0.0009112 ± 0.0001590, *P*=0.0469, **Figure 6E**), *Il-6* (CNV=0.0006228 ± 0.00002669, REP=0.0004757 ± 0.00007688, *P*=0.0010, **Figure 6F**), *Mcp-1* (CNV=0.001929 ± 0.001013, REP=0.0009323 ± 0.0002731, *P*=0.0423, **Figure 6G**) and *Tnfα* (CNV=0.0009293 ± 0.0002358, REP=0.0005273 ± 0.0001732, *P*=0.0072, **Figure 6H**) in the retina-choroid complex tissue. There was a trend for a decrease of *Cxcl10* expression, but this difference did not reach statistical significance (CNV=0.004150 ± 0.001626, REP=0.002558 ± 0.0008236, P=0.1282, **Figure 6I**). To further strengthen the results, Elisa was performed to measure the secreted inflammatory cytokine levels. The results showed that microglial repopulation significantly decreased the secretion of various inflammatory cytokines, including IL-1β (CNV=39.99 ± 9.936, REP=7.327 ± 1.775, *P*<0.0001, **Figure 6J**), IL-6 (CNV=40.44 ± 13.51, REP=7.613 ± 3.720, *P*<0.0001, **Figure 6K**), MCP-1 (CNV=175.6 ± 148.6, REP=25.19 ± 6.079, *P*=0.0079, **Figure 6L**), TNFα (CNV=22.82 ± 5.277, REP=10.39 ± 1.296, *P*<0.0001, **Figure 6M**) and CXCL10 (CNV=0.08257 ± 0.004756, REP=0.07025 ± 0.006213, *P*=0.0002, **Figure 6N**) in the retina-choroid complex tissue.

Altogether, these findings indicate that repopulating microglia produce fewer cytokines involved in the immune response and thereby attenuate the inflammatory response in CNV lesions.

## Discussion

While robust evidence clearly identifies the beneficial effects of microglial repopulation in degenerative neurological diseases (18, 31–33), the contributions of repopulating microglia in the retinal degenerative diseases AMD and the potential mechanisms remain incompletely understood. In this study, we demonstrated that microglial repopulation exacerbated neovascular leakage and angiogenesis formation. We also found that the accumulation of senescent cells in laser sites and treatment with microglial repopulation increased microglial phagocytosis and led to reduced cellular senescence. In addition, new microglia produced less CXCL2 and exhibited lower levels of activation markers than resident microglia, thereby ameliorating leukocyte infiltration and attenuating the inflammatory response in CNV lesions. Our study provides promising insights into the potential of microglial repopulation as a novel, promising therapeutic approach for the treatment of AMD using a mouse model of laser-induced CNV.

Microglia have been implicated to accumulate in the subretinal space, subsequently switching into an activated phenotype and undergoing significant changes in their function in both AMD patients and mouse models (9, 34, 35). These activated microglia cause the excessive release of inflammatory mediators and a prolonged inflammatory response, which may result in the growth of neovascular lesions and further tissue damage (35, 36). As microglial survival and function are critically dependent upon CSF1R, CSF1R inhibition can effectively deplete microglia (12). Withdrawal of CSF1R inhibition results in the rapid repopulation of the whole retina with naïve microglia (14). Now that microglial activation in CNV has been identified as a symptom of inflammatory damage which in turn exacerbates retinal degeneration (9, 10, 36), it is plausible to hypothesize that the replacement of these overactivated microglia with new microglia resembling nonreactive homeostatic microglia may relieve the inflammatory response and promote retinal tissue repair in AMD. As the activated microglia have been reported to recruitment to the laser spots peaked 1 to 4 days after laser injury (37), we establish a corresponding treatment paradigm for microglial elimination and subsequent microglial repopulation in the retina. We observed a better protective effect on microglial repopulation group than depletion group. The beneficial effects of microglia repopulation can be twofold. First, it eliminates hyperactivated microglia in the early stage of treatment, which has similar effects to microglial depletion (38, 39). And second, the newly derived microglia can maintain tissue integrity and promoted neurological recovery via regulating neuroinflammation and secreting neurotrophic factors (38, 39). Furthermore, microglia play integral roles in neural circuit development, neurotransmission, and maintaining brain and retina homeostasis. Indefinite elimination of microglia is unlikely to be clinically feasible. Therefore, repopulation may be a more promising strategy for the treatment of diseases with long-term inflammatory response. As principal innate immune cells of the CNS, microglia is a highly heterogenous cell population. The phenotype of microglia can be protective or pro-inflammatory. The changes in their phenotypes, their loss of protective functions, and their gain of toxic functions are complicated and may differ with the stage and severity of disease (40, 41). Therefore, the optimal timing between microglia depletion and repopulation is important to obtain therapeutic effects. Further studies are warranted to investigate the appropriate time window of microglia repopulation treatment in different ocular diseases.

Although advanced age is the most well-documented risk factor for AMD (1, 3), the ways in which aging impacts AMD progression and how to suppress this influence remain incompletely understood. Notably, previous studies have demonstrated that senescent cells are abundant in human eyes with AMD (42, 43). Accumulating evidence indicates that senescent cells may directly and indirectly damage the local microenvironment, which disrupts the maintenance of retinal homeostasis and exacerbates retinal degeneration (44–46). With age, microglia are presumed to be dystrophic and senescent and show impaired phagocytic function, leading to a buildup of cellular debris and enhanced tissue damage (47, 48). It has been suggested that microglial repopulation treatment serves as an excellent anti-aging intervention for CNS degenerative diseases and the aging brain (21, 32, 33). Casali BT et al. found that repopulated microglia altered the compaction and morphology of plaques, consequently limiting neurotic dystrophy (32). Zhao R et al. reported that microglial repopulation significantly reduced the dendritic abnormalities caused by amyloid-β deposition in Alzheimer’s disease (31). In addition, microglial repopulation reduced lipofuscin deposition, restored aging characteristics and improved cognition in the aging brain (49, 50). Consistent with several previous studies (42, 43), our results showed that cellular senescence characteristics exist in CNV lesions. However, microglial repopulation significantly enhanced microglial phagocytic function and inhibited cellular senescence and reduced CNV size in the laser-induced CNV model, suggesting microglial repopulation as an effective antiaging option for senescence-associated CNV.

CD68 and MHC-II are considered markers of microglia, indicating that microglia are in an activated state (51). In our study, recruited microglia exhibited a strong decrease in the expression of CD68 and MHC-II and the downregulation of cytokine production involved in the immune response pathway in mice receiving microglial repopulation treatment. We also observed reduced expression of inflammatory cytokines and chemokines, including IL-1β, IL-6, MCP-1, TNFα, CXCL10 and CXCL2, in retinal tissue. IL-1β and TNFα are major inflammatory cytokines that serve as biomarkers for choroidal neovascularization (52, 53). IL-6 correlates with CNV size in patients with AMD (54). CXCL2, CXCL10 and MCP-1 have been described to recruit neutrophils, inflammatory monocytes, dendritic cells and other leukocytes to sites of inflammation (55, 56). Therefore, the reduction in the production of these chemokines after microglial repopulation treatment may help to elucidate the mechanism by which repopulated microglia attenuate the inflammatory response, leukocyte infiltration and neovascular formation. A previous study by Liyanage et al. showed the myeloid infiltration into the retina and choroid was composed of inflammatory monocytes, NK cells, dendritic cells and mainly neutrophils in CNV mice (57). In our study, total monocyte, dendritic cells and neutrophils were markedly recruited in the retina-choroid complex of CNV mice, which corroborate with Liyanages’s report. Total monocytes and dendritic cells were accounted for the majority, while very low numbers of neutrophils were observed. An important reason for this different proportion of myeloid result with Liyanages’s study may be the different detection time. Liyanage et al. performed the analysis 3 days after the laser modeling, while we performed our analysis 3 days after the laser modeling. As the primary foot soldier of the immune system, neutrophils accumulated during the initial phases of injury and then decreased with time, while monocyte and dendritic cells expand and peak later (58). Our study and Liyanage’s study suggested that retinal immune reaction and several myeloid cells were involved in the in different phases of CNV, and therefore, immune therapy will show promise in AMD therapy.

In this study, we used a CSF1R antagonist (PLX3397) to eliminate microglia, which can cross the BBB and BRB and deplete microglia in the brain and retina within a few days (15, 23). Despite the numerous strengths of this pharmacological approach, it is crucial to acknowledge the limitations of PLX3397, including relatively low penetrance, potential off-target effects on the tyrosine kinase c-Kit (15) and the potential effects on the peripheral monocyte compartment. Previous studies have yielded inconsistent findings regarding the effect of PLX3397 on blood monocytes (59–61). Although, we found PLX3397 treatment did not lead to a notable reduction of blood monocyte in CNV mice (data not shown). This was one of the limitations of the current study, and the effect of PLX3397 and subsequent withdraw on blood cell phenotyping warrants further investigation. Genetic interventions have also been utilized to enable microglial repopulation, such as Cx3cr1CreERT2/+Csf1r+/fl and toxin-based models. While transgenic models may offer higher specificity, their application produces a strong neuroinflammatory response or disrupts microglial homeostasis (62, 63). Consequently, short-term PLX3397 treatment followed by recovery upon withdrawal seems to be a more clinically feasible option for the treatment of AMD. Although our results reveal the protective effect of microglial repopulation in CNV lesions, further investigations employing more specific drugs, such as PLX5622, are needed to verify our findings.

**Figure 7:**
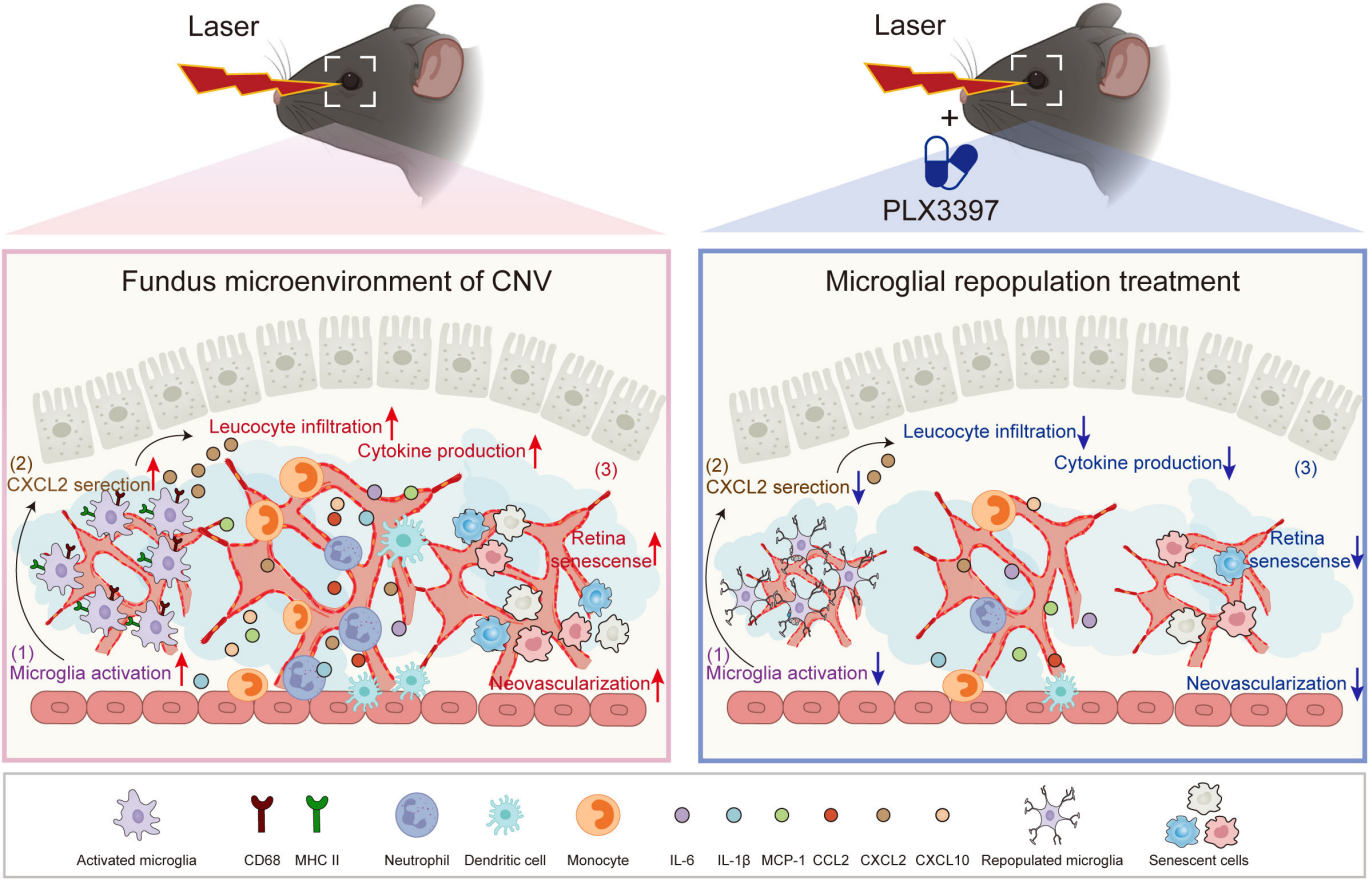
The proposed working model. In the CNV eyes, recruited microglia become activated and provoke the amplification of inflammation, contributing to the progression of CNV pathology. Following microglial elimination with PLX3397 and subsequent withdrawal, microglial repopulation restores the primed microglia to homeostatic microglia, restricts inflammation and cellular senescence, thereby attenuating choroidal neovascularization.

In conclusion, our work demonstrates that microglial repopulation in the retina attenuates inflammation and choroidal neovascularization, suggesting that it may be a promising strategy for the treatment of AMD **Figure 7**.

## Data availability statement

The original contributions presented in the study are included in the article. Further inquiries can be directed to the corresponding authors. All sequencing data have been submitted to the NCBI Sequence Read Archive (SRA; http://www.ncbi.nlm.nih.gov/bioproject/1100172) under accession number PRJNA1100172.

## Ethics statement

The animal study was approved by the Institutional Animal Care and Use Committee of Shenzhen TopBiotech Corporation. The study was conducted in accordance with the local legislation and institutional requirements.

## Author contributions

YS: Writing – review & editing, Writing – original draft, Software, Project administration, Methodology, Funding acquisition, Formal Analysis, Data curation. YL: Writing – original draft, Methodology, Investigation. TL: Writing – original draft, Methodology, Investigation. YC: Writing – original draft, Resources, Funding acquisition, Data curation. FW: Writing – original draft, Resources, Data curation. ZZ: Writing – review & editing, Validation, Supervision. WZ: Writing – review & editing, Formal Analysis. JL: Writing – review & editing, Supervision, Resources, Conceptualization.
